# Defining *Porphyromonas gingivalis* strains associated with periodontal disease

**DOI:** 10.1038/s41598-024-56849-x

**Published:** 2024-03-14

**Authors:** Vijaya Murugaiyan, Simran Utreja, Kathleen M. Hovey, Yijun Sun, Michael J. LaMonte, Jean Wactawski‑Wende, Patricia I. Diaz, Michael J. Buck

**Affiliations:** 1 Department of Biochemistry, Jacobs School of Medicine and Biomedical Sciences, University at Buffalo, Buffalo, NY USA; 2 Department of Epidemiology and Environmental Health, School of Public Health and Health Professions, University at Buffalo, Buffalo, NY USA; 3 Department of Microbiology and Immunology, Jacobs School of Medicine and Biomedical Sciences, University at Buffalo, Buffalo, NY USA; 4 UB Microbiome Center, University at Buffalo, Buffalo, NY USA; 5 Department of Oral Biology, School of Dental Medicine, University at Buffalo, Buffalo, NY USA; 6 Department of Biomedical Informatics, Jacobs School of Medicine and Biomedical Sciences, University at Buffalo, Buffalo, NY USA

**Keywords:** Microbiology, Molecular biology, Dental diseases, Neurological disorders

## Abstract

*Porphyromonas gingivalis,* a Gram-negative anaerobic bacterium commonly found in human subgingival plaque, is a major etiologic agent for periodontitis and has been associated with multiple systemic pathologies. Many *P. gingivalis* strains have been identified and different strains possess different virulence factors. Current oral microbiome approaches (16S or shotgun) have been unable to differentiate *P. gingivalis* strains. This study presents a new approach that aims to improve the accuracy of strain identification, using a detection method based on sequencing of the intergenic spacer region (ISR) which is variable between *P. gingivalis* strains. Our approach uses two-step PCR to amplify only the *P. gingivalis* ISR region. Samples are then sequenced with an Illumina sequencer and mapped to specific strains. Our approach was validated by examining subgingival plaque from 153 participants with and without periodontal disease. We identified the avirulent strain ATCC33277/381 as the most abundant strain across all sample types. The W83/W50 strain was significantly enriched in periodontitis, with 13% of participants harboring that strain. Overall, this approach can have significant implications not only for the diagnosis and treatment of periodontal disease but also for other diseases where *P. gingivalis* or its toxins have been implicated, such as Alzheimer's disease.

## Introduction

Periodontitis is among the most common infections in the United States, with an estimated prevalence of 42.2% for adults aged 30 years or older, and 7.8% of individuals experience severe periodontitis^1^. Untreated periodontitis can lead to impaired oral function with eventual tooth loss and an overall reduced quality of life^2–4^. Periodontitis has been associated with a variety of systemic disorders such as cardiovascular disease, diabetes, Alzheimer's disease, respiratory tract infection, and adverse pregnancy outcomes^5^.

Periodontitis has been characterized as a dysbiotic disease owing to a shift in the subgingival microbial communities that colonize the periodontal pockets from a predominantly Gram-positive aerobic bacteria, to a dominance of Gram-negative anaerobes. The most notable are the so-called “red-complex” bacteria: *Porphyromonas gingivalis, Tannerella forsythia, and Treponema denticola*^6^. A growing body of literature suggests *P. gingivalis*, the keystone pathogen in chronic periodontitis, has implications in the onset of different systemic pathologies, including rheumatoid arthritis, cardiovascular disease, and neurodegenerative pathologies^7,8^. A recent study provides evidence of presence of gingipains, a toxic protease produced by *P. gingvalis*, in the brain of Alzheimer's patients suggesting *P. gingvalis* may play a critical role in the etiology of Alzheimer's disease^9^.

*P. gingvalis* is found in healthy individuals as well as in patients with chronic periodontal disease^10–13^. Several characterization studies have examined the genetic diversity among *P. gingivalis* strains and their varying potency to cause periodontal diseases. Approaches including multilocus enzyme electrophoresis, RAPD fingerprinting, and MLS analysis have separated *P. gingivalis* into 41–73 different strains^14–17^.

Currently, there are 67 unique *P. gingivalis* genomes in the NCBI database. Experimentally, strains of *P. gingivalis* vary in the manifestation of measured virulence traits^10,18^. For example, subcutaneous injection of certain strains, including W83, can cause spreading and ulcerating lesions at distant sites whereas others produce only small, localized abscesses^19^. Platelet activation, collagenase activity, and macrophage avoidance have all been shown to vary by strain^20–22^.

Current microbiome approaches involve sequencing of the hypervariable regions of the 16S rRNA gene or shotgun sequencing of all isolated DNA. Both approaches are limited in their ability to detect bacterial subspecies or strains. For example, although 16S microbiome experiments have revealed the diversity of the microbiota and have uncovered a connection with human health and disease^23–27^, many bacteria cannot be identified to the species level, and 16S sequencing approaches completely miss strain differences. While shotgun sequencing is commonly utilized for gut microbiome research, it is not as practical for analyzing the oral microbiome. This is because a substantial portion (~ 90%) of the DNA obtained from the mouth is from the human host^28^. Consequently, the standard approach to shotgun sequencing can be expensive when attempting to identify highly diverse and complex bacterial communities with high accuracy^29^. Additionally, when conducting strain-level analysis, a considerably greater depth of sequencing is required, and current computational techniques for detecting strains have their limitations^30^. When examining subgingival plaque samples, the abundance of *P. gingivalis* can range from 5 to 0.1% in individuals with moderate to severe periodontal disease^31^. As a result, for any large-scale project, the cost of sequencing at a depth required for strain identification would be cost-prohibitive.

Thus, to determine *P. gingivalis* strains cost-effectively, we have adapted previous approaches examining the intergenic spacer region (ISR)^17,32,33^. The ISR is between the small (16S) and large (23S) ribosomal subunit genes and is highly variable among strains^34–36^. For this study, we identified an information-rich region located within the *P. gingivalis* ISR and generated specific primers for sequencing that only target *P. gingivalis* and flank a region containing strain-identifying differences. We validated our approach by examining subgingival plaque from 153 participants with and without periodontal disease and demonstrated a significant association of the W83 strain with disease severity.

## Results

### Specificity, sensitivity and reproducibility of *P. gingivalis* ISR-specific primer

**Figure 1:**
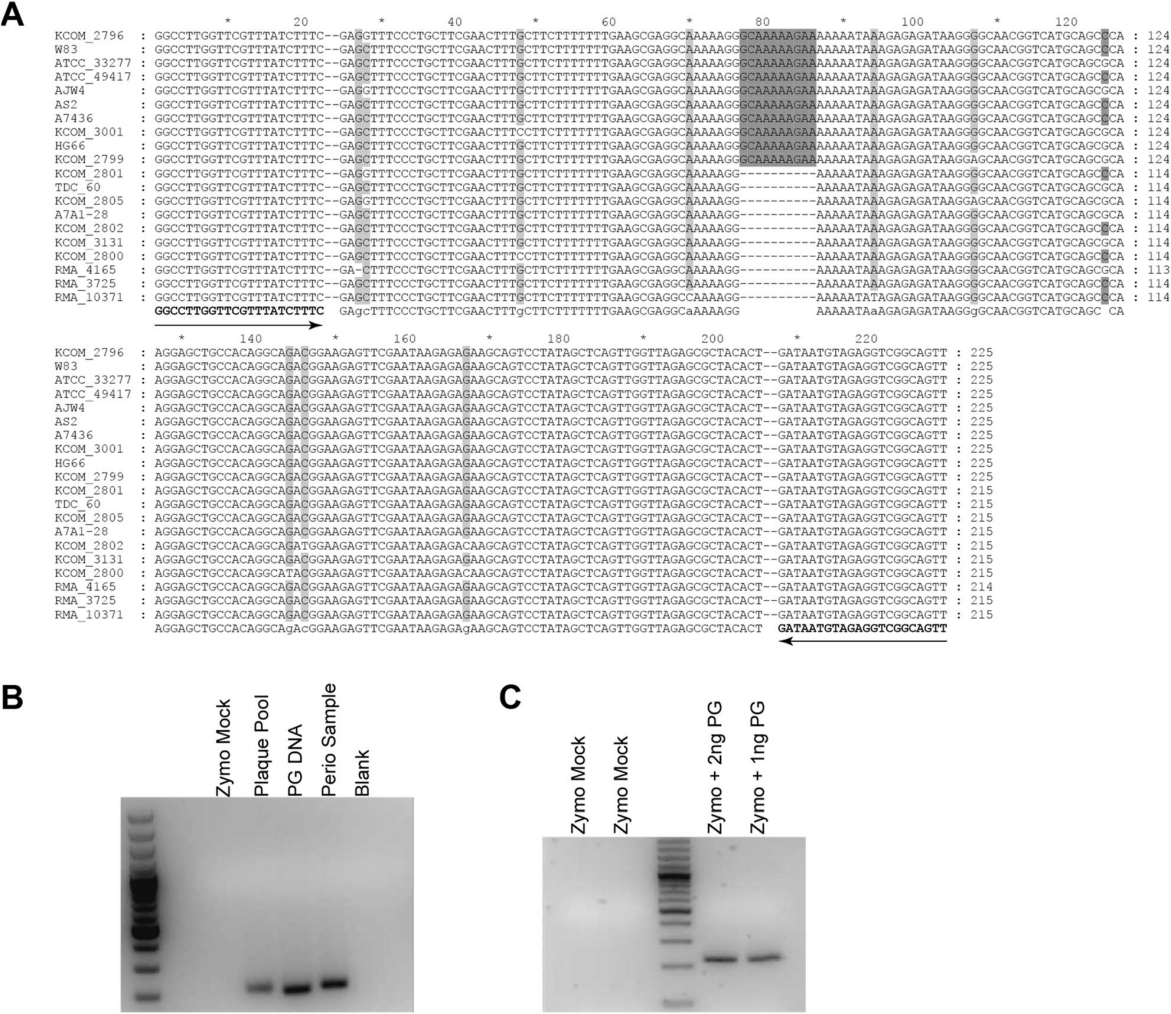
Creating *P. gingivalis* specific primers. (**A**) Alignment of representative *P. gingivalis* strain ISR regions. Primer locations are indicated. (**B**) PCR conditions were optimized to amplify *P. gingivalis* ISR regions without targeting other bacteria. (**C**) PCR conditions allow *P. gingivalis* amplification in mixed samples. Zymo Mock contains DNA from *Listeria monocytogenes, Pseudomonas aeruginosa, Bacillus subtilis, Escherichia coli, Salmonella enterica, Lactobacillus fermentum, Enterococcus faecalis, and Staphylococcus aureus*; Plaque Pool, pooled subgingival plaque from multiple participants (positive control for sequencing batches); Pg. DNA, *P. gingivalis* strain ATCC33277. Perio Sample, subgingival plaque from a participant with severe periodontal disease. Amplicons were visualized in 2% agarose gel electrophoresis with ethidium bromide staining.

Available ISR sequences for *P. gingivalis* were aligned to identify 200–250 bp regions containing the highest number of differences between strains which are flanked by conserved sequences for PCR amplicons (Fig. 1). The conserved primer sequences were then blasted to the NCBI database to test identity to non- *P. gingivalis* bacteria. Two sets of primers were identified with a single set described below as being the most informative for strain identification. Specificity of the *P. gingivalis* ISR-specific primers were determined with subgingival plaque from a participant with severe periodontal disease, positive controls (*P. gingivalis* strain ATCC33277, pooled subgingival plaque from multiple participants), and negative controls (Zymo mock DNA, Microbial DNA-free water) (Fig. 1B). The primers specific for *P. gingivalis* amplified *P. gingivalis* DNA (~ 215 to 250 bp) in the participant sample and positive controls, whereas PCR products were not amplified in negative controls. Sensitivity of the *P. gingivalis* ISR-specific primer was determined with and without spiking of *P. gingivalis* strain ATCC33277 (1 ng and 2 ng) in Zymo mock DNA (Fig. 1C). The sensitivity of ISR-specific primers were confirmed by amplifying *P. gingivalis* DNA only in the spike-in reaction and verifies that amplification of *P. gingivalis* DNA is not inhibited by the presence of other microbial DNA in a reaction.

### Identifying *P. gingivalis* strains in sub-gingival plaque

To examine *P. gingivalis* strains associated with periodontal disease we examined subgingival plaque samples from the Buffalo Osteoporosis and Periodontal Disease (OsteoPerio) Study^37^. The OstroPerio study is a prospective cohort study that was established to explore risk factors for the development and progression of osteoporosis and periodontal disease in postmenopausal women. The subgingival microbiome was previously examined using 16S rRNA gene sequencing^31^. In total 161 subgingival plaque samples were examined with 12 plaque pools, 2 positive controls, and 6 negative controls.

**Figure 2:**
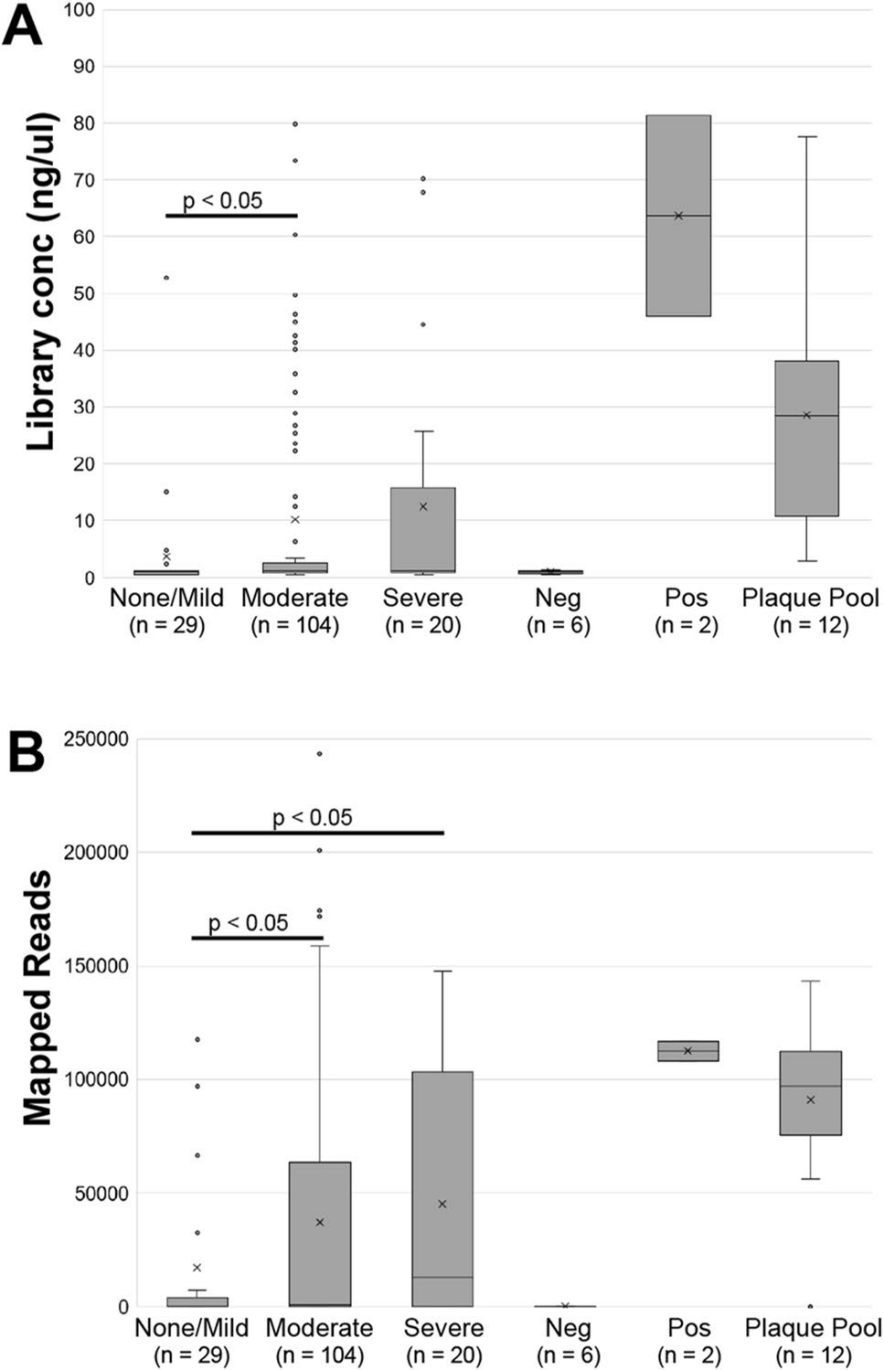
ISR libraries and number of mapped reads. (**A**) DNA concentrations after next-generation sequencing library formation with two rounds of PCR. (**B**) Numbers of reads that mapped to the *P. gingivalis* ISR database. The sample periodontal classification (none/mild, moderate, severe) with the number of samples is shown for each group. Neg, batch-specific negative control (extraction buffer, PCR blank); Pos, positive control with ATCC33277 DNA; Plaque pool, DNA isolated from a mixture of subgingival plaques from a group of people (positive control).

*P. gingivalis* ISR specific amplicons were generated using two-step PCR, similar to 16S analysis (see “Methods”)^38^. DNA concentration of each ISR sequencing library was quantified before sequencing. DNA concentration vs. each sample type (Periodontal health status—None/Mild, Moderate and Severe, negative and positive controls) is illustrated in Fig. 2A. *P. gingivalis* DNA concentration was significantly greater in the moderate and severe periodontal disease groups than in the none/mild group, consistent with increased abundance of *P. gingivalis* in moderate and severe disease groups. An insignificant amount of DNA was detected in the negative controls (extraction buffer, Zymo mock DNA, Microbial DNA-free water). After sequencing, quality filtering, and pair-read merging, sequence reads were mapped to a custom-built ISR database containing unique *P. gingivalis* reference sequences using DADA2^39^. The number of reads mapping was consistent with sequencing library concentration with moderate and severe periodontal disease groups having a higher number of *P. gingivalis* reads (Fig. 2B).

### Detection of *P. gingivalis* strains

**Figure 3:**
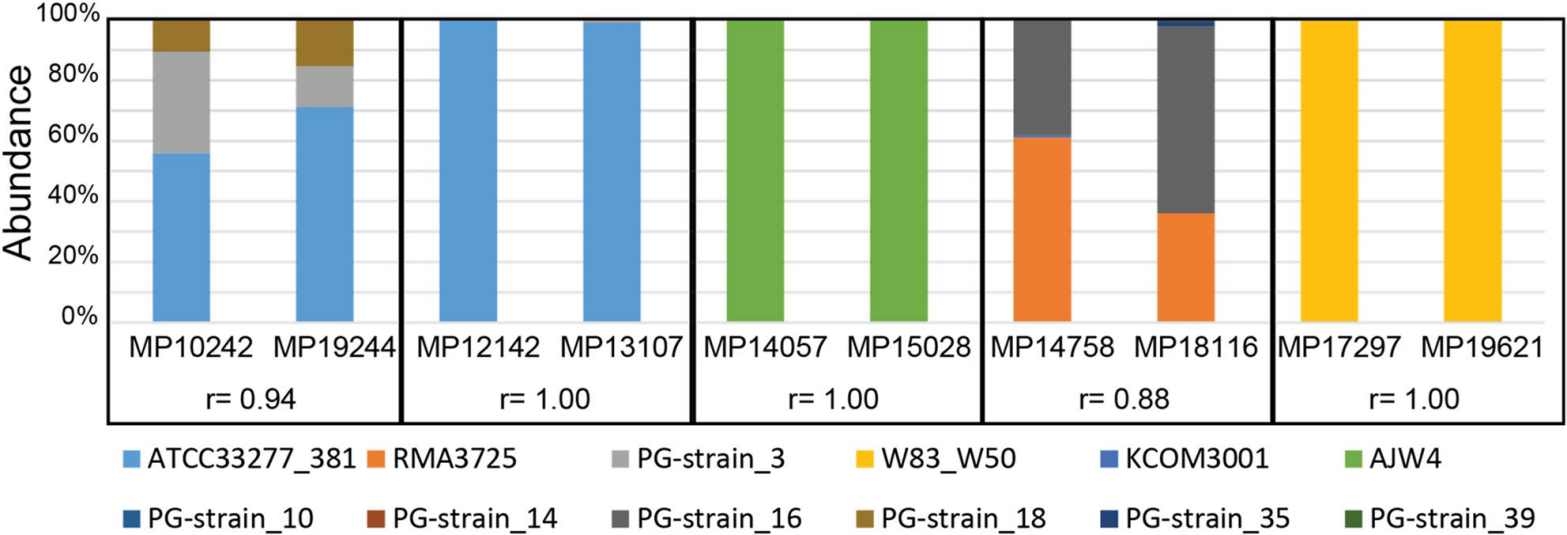
Technical replicates are reproducible. Relative abundance for 5 replicate samples, with the Pearson correlation (r) between replicates.

There were 139 unique sequences of *P. gingivalis* ISR. Each possible strain is named by 100% identity to available ISR sequences. When multiple strains have the same ISR sequence all names are included. For example, the related strains W83 and W50 have identical ISR sequences for this region and cannot be separated. Many identified ISR sequences are novel and named PG-Strain #. Each novel strain has been further characterized by sequence similarity (Supplemental Dataset 1). Two positive control samples contained strain ATCC33277. For these samples, 99.99% (108,163/108,177) or 100% (116,901/116,901) of sequenced reads mapped to ATCC33277. In addition, 5 replicates were included where the plaque sample was split before DNA extraction. The replicates experiments were very similar to each other, demonstrating the reproducibility of our approach (Fig. 3).

**Figure 4:**
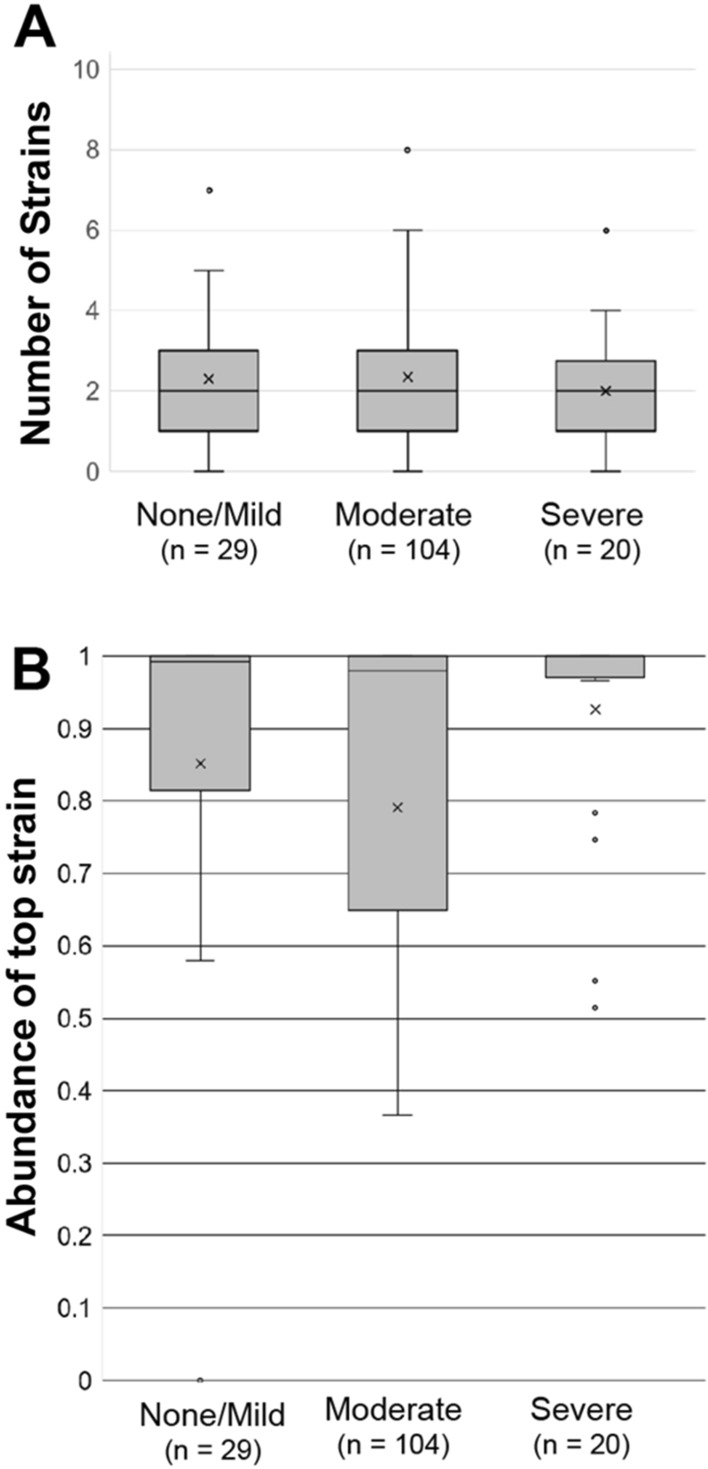
Number of strains and abundance (**A**) Number of detected strains in each sample. (**B**) Frequency of the most abundant strain in each sample.

Most participant samples mapped to two different *P. gingivalis* strains (Fig. 4A), consistent with previous findings^17^. Alpha diversity (Shannon, Chao1) are similar across periodontal disease status (Supplemental Fig. 1). To further understand the distribution of *P. gingivalis* strains in each sample we determine the frequency of the top strain in each sample (Fig. 4b). For most participant samples regardless of disease state, the top strain represents > 90% of all *P. gingivalis* sequence reads.

### W83/W50 is associated with periodontal disease

To determine if specific strains are associated with periodontal disease, we grouped our samples into two groups None/Mild and Moderate/Severe and filtered the ISR sequences removing strains with low abundance or ones appearing in a single sample (see “Methods”). In total 18 *P. gingivalis* strains were examined after filtering (Table 1).

**Table 1:** *P. gingivalis* strains across different categories of periodontal disease.

Strain label	Periodontal disease category^a^				Whole-mouth mean					
	None/mild (n = 21)		Moderate/severe (n = 104)		FDR		PD		CAL	
	Mean^b^ (SE)	Freq^c^	Mean^b^ (SE)	Freq^c^	P value^d^	q-value^e^	*r*^f^	P value^g^	*r*^f^	P value^g^
W83_W50	0 (0)	0	1.45 (0.38)	0.13	0.0003	0.005	0.142	0.11	0.096	0.29
PG-strain_10	0 (0)	0	0.33 (0.2)	0.02	0.09	0.43	0.076	0.4	0.152	0.09
PG-strain_45	0 (0)	0	0.18 (0.1)	0.01	0.09	0.43	0.271	0.002	0.23	0.01
KCOM2798	0.21 (0.21)	0.05	0.77 (0.26)	0.11	0.10	0.43	0.052	0.56	0.031	0.73
PG-strain_16	0 (0)	0	0.23 (0.17)	0.02	0.16	0.43	− 0.094	0.3	− 0.051	0.58
PG-strain_39	0 (0)	0	0.14 (0.1)	0.01	0.16	0.43	− 0.075	0.4	− 0.025	0.78
PG-strain_43	0 (0)	0	0.12 (0.09)	0.01	0.17	0.43	− 0.017	0.85	− 0.066	0.46
KCOM3001	0.28 (0.28)	0.05	0.72 (0.27)	0.06	0.27	0.61	− 0.134	0.14	− 0.064	0.48
ATCC33277_381	6.38 (0.96)	0.71	5.34 (0.52)	0.54	0.34	0.66	− 0.028	0.75	− 0.014	0.88
PG-strain_18	0.4 (0.23)	0.1	0.64 (0.17)	0.13	0.40	0.66	− 0.071	0.43	− 0.067	0.46
TDC60_KCOM3131	0.3 (0.3)	0.05	0.61 (0.24)	0.05	0.43	0.66	− 0.057	0.53	− 0.095	0.29
RMA3725	1.46 (0.78)	0.19	2.16 (0.43)	0.2	0.44	0.66	− 0.07	0.44	0.007	0.94
KCOM2796	0.73 (0.73)	0.05	0.33 (0.16)	0.04	0.60	0.82	− 0.024	0.79	− 0.032	0.72
PG-strain_8	0.29 (0.29)	0.05	0.4 (0.18)	0.03	0.76	0.85	0.034	0.71	0.1	0.27
AJW4	0.64 (0.46)	0.1	0.78 (0.29)	0.06	0.78	0.85	− 0.11	0.22	− 0.011	0.9
PG-strain_3	2.21 (0.97)	0.29	2.48 (0.43)	0.28	0.80	0.85	0.114	0.21	0.108	0.23
PG-strain_14	0.17 (0.17)	0	0.23 (0.16)	0.01	0.80	0.85	− 0.004	0.96	− 0.009	0.92
PG-strain_9	0.51 (0.51)	0.05	0.44 (0.22)	0.05	0.90	0.90	− 0.061	0.5	− 0.035	0.7

CAL, clinical attachment level; PD pocket depth.

^a^Defined according to Centers for Disease Control and Prevention/American Academy of Periodontology criteria^40^.

^b^Mean centered log_2_-ratio transformed strain.

^c^Frequency of strain detected with at least 1% of reads.

^d^P value comparing None/Mild to Moderate/Severe.

^e^q-value for Benjamini Hochberg FDR.

^f^Pearson product-moment correlation coefficient.

^g^P value for correlation coefficient.

The most abundant strain across all samples is the avirulent *P. gingivalis* strain ATCC33277_381 which was found in both the none/mild (CLR 6.38) and moderate/severe groups (CLR 5.34). The majority 15/18 strains are more abundant in Moderate/Severe cases. Only strain W83_W50 was significantly enriched in the Moderate/Severe category after correcting for multiple testing. W83_W50 was only detected in the moderate/severe group, with 13% of participants harboring that strain. When we compared the abundance levels using centered log2-ratio (CLR)-transformed read counts, strain W83 was significantly enriched in the moderate/severe group (p = 0.0003; q = 0.005). Strain W83 was previously shown by heteroduplex typing to be strongly associated with periodontitis^41^. Multiple studies have shown that strain W83 was more virulent than other *P. gingivalis* strains^19,42–44^.

We next evaluated the linear correlations for each CLR mean strain with the whole-mouth clinical measurements for mean pocket depth (PD) and clinical attachment level (CAL) (Table 1). Correlations ranged from − 0.134 to 0.142 for PD and from − 0.095 to 0.152 for CAL. After multiple testing correction none of correlations are significant.

## Discussion

Determining *P. gingivalis* strains with current microbiome methods has been limited. There are two major issues which needed to be addressed. First, abundance of *P. gingivalis* in the total DNA pool is very low. For oral samples, the majority of isolated DNA is from the human host and the abundance of *P. gingivalis* in the microbiome is only 5–0.1% for people with moderate to severe periodontal disease^31^. *P. gingivalis* is also detectable in healthy patents as shown in our results and by previous studies^10–13^. Second, strain identification requires strain specific sequence differences in a region which was sequenced in all the samples. Our approach addresses both concerns by amplifying a *P. gingivalis* specific ISR sequence at a region with strain defining sequence variability. In addition, our approach can detect low levels of *P. gingivalis* in healthy participants allowing a robust examination of strains in relationship to various medical conditions.

In this study we identified an information-rich region located within the *P. gingivalis* ISR that can segregate *P. gingivalis* strains in a cost-effective manner. To validate our ISR sequencing approach we examined subgingival plaque from 153 participants. Only the ISR region identified as W83_W50 was significantly associated with periodontal disease. W83 has previously been shown to be associated with periodontitis^41^ and multiple studies have shown W83 as being more virulent than other strains^19,42–44^. Several strains were identified only in the Moderate/Severe category, but due to the limited sample size were not significant in this study. Strain ATCC33277 was the most abundant strain identified in this study and there was a slight enrichment for the None/Mild category. ATCC33277 has been proposed to be healthier strain with reduced virulence characteristics^45,46^.

While our approach is promising, it does have some limitations that should be taken into consideration. The first limitation is that a significant number of *P. gingivalis* strains have identical sequences within our targeted ISR region. Consequently, our current approach cannot differentiate between these strains. Identification of additional primer sets located in virulence genes would strength this approach and facilitate the characterization of unknown ISR strain types. Secondly, our ability to identify each strain is dependent on matching sequences to a database of characterized strains. However, this limitation will improve as more *P. gingivalis* strains are sequenced and characterized over time. Lastly, it is worth noting that all low complexity amplicon sequencing approaches that use Illumina sequencing require a large spike-in of DNA. This is because almost all bases on the flow cell have the same base call, leading to issues with adjusting the fluorescence intensity. To address this issue, our primers can include additional dephasing bases that enable clustering at higher density^47^ or alternatively, samples can be multiplexed with other high complexity experiments.

## Methods

### Participants

The present study included 153 postmenopausal women enrolled in the Buffalo Osteoporosis and Periodontitis (OsteoPerio) Study, which is an ancillary study conducted at the Buffalo (NY) clinical center of the Women’s Health Initiative Observational Study (WHI OS). Each participant in this study had a complete clinical oral examination^37^. The mean pocket depth (PD) and clinical attachment level (CAL) measurements define based on the criteria of Centers for Disease Control and Prevention (CDC)/American Academy of Periodontology (AAP) was used to determine the clinical periodontal disease status as none, mild, moderate, and severe^40^. Subgingival plaque samples were collected in Ringer’s solution using fine paper points and frozen immediately at − 80 °C^48^. All participants provided informed consent, and the study protocol was approved by the University at Buffalo’s Health Sciences Institutional Review Board. All experiments were in agreement with relevant guidelines regarding Human Subjects Research.

### Bacterial genomic DNA extraction

Metagenomic DNA was isolated from subgingival plaque samples using an the QIAsymphony SP automated system as described previously^31,49,50^. Before DNA extraction, an enzymatic pretreatment was performed for more efficient isolation of Gram-positive bacteria. 500 μl of subgingival plaque samples were pelleted by centrifugation at 5000×*g* for 10 min, at room temperature, and the pellet was resuspended in 300 μl lysis solution (20 mg/ml lysozyme in 20 mM Tris–HCl, pH 8.0; 2 mM EDTA; 1.2% Triton X-100) and incubated at 37 °C for 30 min. DNA extraction and purification were done according to the Qiasymphony DSP Virus /Pathogen Kit Instructions for Use (Handbook) and the QIAsymphony SP Protocol Sheet for the Complex200_V6_DSP protocol (“Complex200_V6_DSP”). After DNA purification, samples were eluted in a 96 well plate (Qiagen, Valencia, CA). Each plate had 77 subgingival plaque samples, duplicate of blank extraction controls, and 6 plaque pool as a positive control. 60ul of DNA was eluted from each sample. Extracted DNA were quantified with the Quant-iT™ High-Sensitivity dsDNA Assay Kit (Invitrogen).

### ISR amplicon sequencing library preparation

Metagenomic DNA from each sample was used to prepare 16 s/23 s Intergenic spacer region (ISR) amplicon sequencing libraries targeting the IRS regions of *P. gingivalis* bacteria. Amplicon libraries were prepared by using a two-step protocol in which the region of interest is first amplified with ISR region specific primers (Forward TCGTCGGCAGCGTCAGATGTGTATAAGAGACAG-GGCCTTGGTTCGTTTATCTTTC; Reverse GTCTCGTGGGCTCGGAGATGTGTATAAGAGACAG-AACTGCCGACCTCTACATTATC) Then dual indices are added through a second PCR. Zymo mock DNA (ZymoBIOMICS Microbial Community DNA Standard, Zymo Research, USA) and Microbial DNA-free water (Microbial DNA-free PCR water, Qiagen Inc., USA) as negative control, and *P. gingivalis* strain ATCC3372 as a positive control was used during the amplification process. Amplicon amplification was performed using thermocycling with 10 μl of genomic DNA, 2 μl of amplicon PCR forward primer (2.5 μM), 2 μl of amplicon PCR reverse primer (2.5 μM), and 12.5 μl of 2 × KAPA HiFi HotStart Ready Mix (Kapa Biosystems) at 95 °C initial denaturation for 3 min, followed by 30 cycles of 95 °C for 30 s, 61.4 °C for 30 s, and 72 °C for 30 s, and a final extension at 72 °C for 5 min. According to the manufacturer’s protocol, reactions were cleaned up with Agencourt AMPure XP beads (Beckman Coulter Genomics). Attachment of dual indices with Illumina sequencing adapters were performed using 5 μl of amplicon PCR product DNA, 5 μl of Illumina Nextera XT Index Primer 1 (N7xx), 5 μl of Nextera XT Index Primer 2 (S5xx), 25 μl of 2 × KAPA HiFi Hot Start Ready Mix, and 10 μl of Microbial DNA-free PCR water, with thermocycling at 95 °C for 3 min, followed by 8 cycles of 95 °C for 30 s, 55 °C for 30 s, and 72 °C for 30 s, and a final extension at 72 °C for 5 min. ISR amplicon libraries were purified with Agencourt AMPure XP beads and quantified with Quant-iT™ High-Sensitivity dsDNA Assay Kit (Invitrogen) and the KAPA Library Quantification Kit (KAPABIOSYSTEMS). Library quality control was performed with the Agilent Technologies 2100 Bioanalyzer to ascertain the quality and average size distribution. Libraries were normalized and pooled to 4 nM based on Quant-iT qualified values. Pooled samples were denatured and diluted to a final concentration of 10 pM with a 20% PhiX (Illumina) control. All sequencing was performed on a single flow-cell using the Illumina Miseq with 2 × 300. All samples were multiplexed and sequenced at 1–5 ratio with shotgun microbiome samples, to reduce the effect of the low-complexity library.

### Data analysis

Available genomes for the *P. gingivalis* bacteria were downloaded from NCBI’s GenBank database. The human oral ISR database was created by extracting ISR sequences with ISR-flanking primers using BLASTN application 2.5.0 from available genomes for all the Porphyromonas gingivalis strains.

The Divisive Amplicon Denoising Algorithm 2 (DADA2—available at https://github.com/benjjneb/dada2) microbiome pipeline used to distinguish the microbial communities by sequence variants differing by as little as one nucleotide present in the data, as amplicon sequence variants (ASVs)^51^. In the first step, both forward and reverse primers were identified in paired-end ISR amplicon sequencing reads and removed using Cutadapt. Read qualities were determined by the frequency of each quality score at each base position using the dada2 function PlotQualityProfile. Reads with a quality score below 30 were removed from further analysis. Filter and trim were performed on the quality-filtered reads using dada2 function filterAndTrim with standard parameters (a maximum number of ambiguous nucleotides (MaxN = 0), reads with expected errors (maxEE = 2)). The sequencing error rates were estimated from all samples as a pool (dada2 function learnErrors), and the filtered sequences were then dereplicated (dada2function derepFastq) to generate unique sequences. These unique sequences were then processed using the DADA2 algorithm (dada2 function mergePairs and makeSequenceTable) to infer exact amplicon sequence variants (ASVs). Chimeric sequences were removed (dada2 function removeBimeraDenovo), a sample vs. ISR ASV table (OTU-tables) was generated (phyloseq function otu_table), which was used for mapping against the custom-built ISR database containing unique *P. gingivalis* reference sequences to identify the *P. gingivalis* strains. Novel ISR sequences without an exact match will be given a default name and searched by BLAST to determine the closest strains.

Each ISR ASV was normalized using the centered log (2)-ratio (CLR) transformation, which will help account for the compositional data structure, reduce the likelihood of spurious correlations, and enhance the meaningfulness of comparison^31,52^. Correlations between technical replicates were calculated using the Pearson correlation coefficient. Results of the frequency *P. gingivalis* strains are presented as mean ± standard error (SE) for different periodontal health statuses with corrected p-values with discrete false-discovery rate correction for multiple testing. Linear relationships between CLR abundance of *P. gingivalis* strain and mean PD, and CAL parameter measurements were evaluated using Pearson correlations. Alpha diversity and univariant strain enrichment tests were performed with online resource MicrobiomeAnalyst^53^.

### Supplementary Information

Supplementary Information 1.

Supplementary Information 2.

## Data Availability

The resulting sequencing data has been uploaded to the NCBI Sequence Read Archive (SRA) database with BioProject ID # PRJNA982061.
