# Supplementary material for: Defining Porphyromonas gingivalis strains associated with periodontal disease
Source: Sci Rep. 2024 Mar 14;14:6222. doi: 10.1038/s41598-024-56849-x (PMC10940620; doi:10.1038/s41598-024-56849-x)
Supplement: Supplementary file 2 — Supplementary Information 2. [file 41598_2024_56849_MOESM2_ESM.pdf]

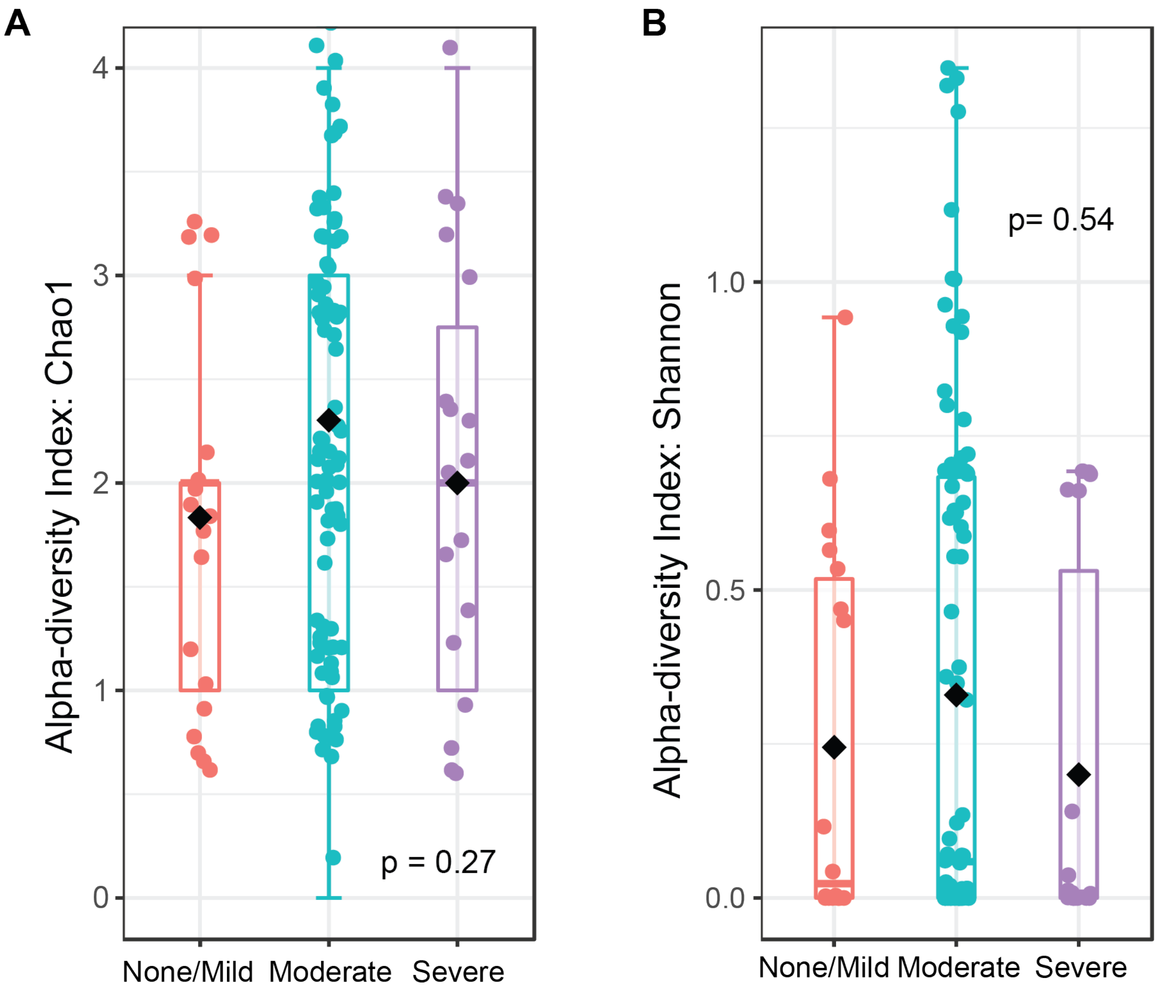

Supplementary Figure 1. Alpha diversity across periodontitis grades. (A) Chao1 index and the (B) Shannon index for each periodontal classification (none/mild, moderate, severe).

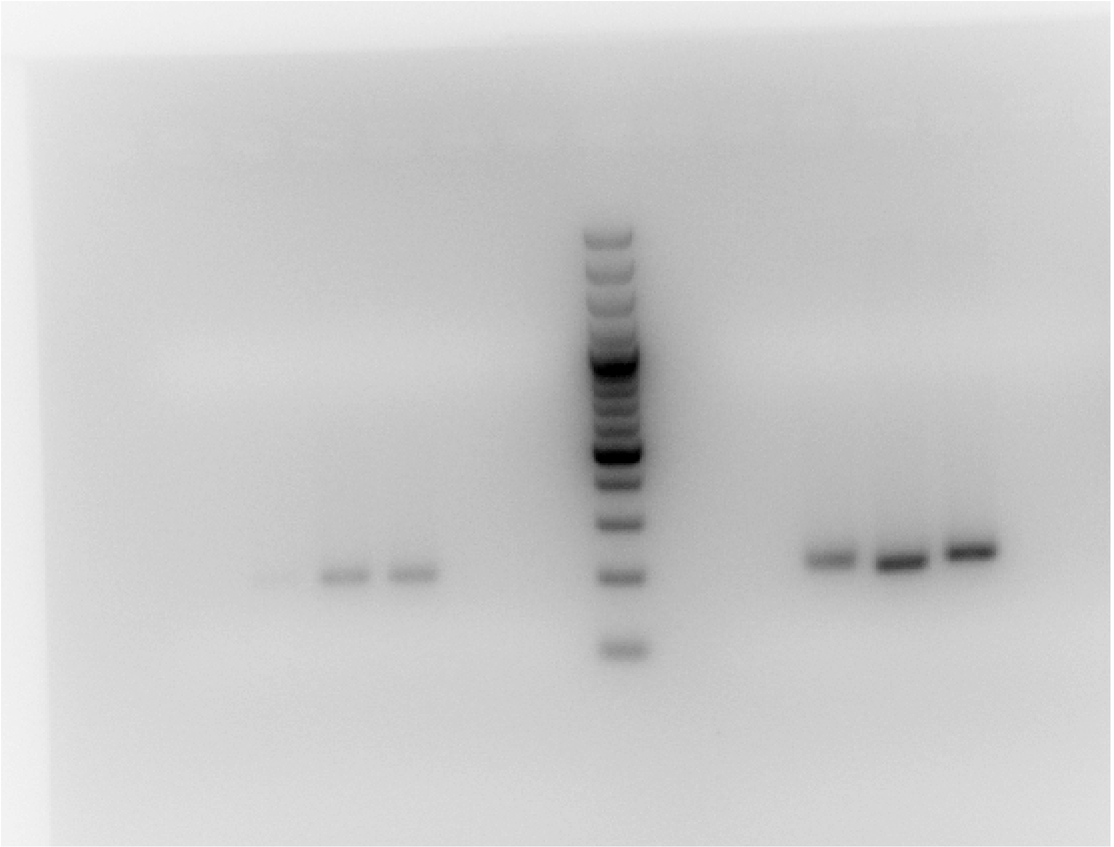

Supplementary Figure 2. Full gel image for Figure 1B

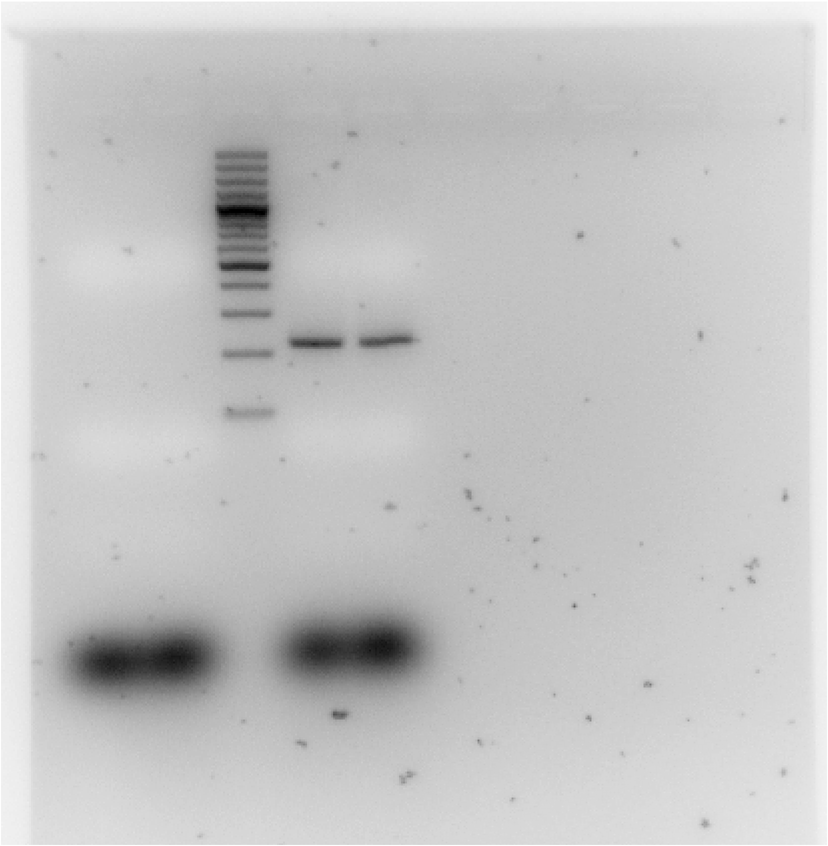

Supplementary Figure 3. Full gel image for Figure 1C
